# Choosing important health outcomes for comparative effectiveness research: An updated systematic review and involvement of low and middle income countries

**DOI:** 10.1371/journal.pone.0190695

**Published:** 2018-02-13

**Authors:** Katherine Davis, Sarah L. Gorst, Nicola Harman, Valerie Smith, Elizabeth Gargon, Douglas G. Altman, Jane M. Blazeby, Mike Clarke, Sean Tunis, Paula R. Williamson

**Affiliations:** 1 MRC North West Hub for Trials Methodology Research, Department of Biostatistics, University of Liverpool, Liverpool, United Kingdom; 2 School of Nursing and Midwifery, Trinity College Dublin, Dublin, Ireland; 3 Centre for Statistics in Medicine, Nuffield Department of Orthopaedics, Rheumatology and Musculoskeletal Sciences, University of Oxford, Oxford, United Kingdom; 4 MRC ConDuCT II Hub for Trials Methodology Research, Population Health Sciences, Bristol Medical School, University of Bristol, Bristol, United Kingdom; 5 Centre for Public Health, Queen’s University Belfast, Belfast, United Kingdom; 6 Center for Medical Technology Policy (CMTP), World Trade Center Baltimore, Baltimore, MD, United States of America; Johns Hopkins University Bloomberg School of Public Health, UNITED STATES

## Abstract

**Background:**

Core outcome sets (COS) comprise a minimum set of outcomes that should be measured and reported in all trials for a specific health condition. The COMET (Core Outcome Measures in Effectiveness Trials) Initiative maintains an up to date, publicly accessible online database of published and ongoing COS. An annual systematic review update is an important part of this process.

**Methods:**

This review employed the same, multifaceted approach that was used in the original review and the previous two updates. This approach has identified studies that sought to determine which outcomes/domains to measure in clinical trials of a specific condition. This update includes an analysis of the inclusion of participants from low and middle income countries (LMICs) as identified by the OECD, in these COS.

**Results:**

Eighteen publications, relating to 15 new studies describing the development of 15 COS, were eligible for inclusion in the review. Results show an increase in the use of mixed methods, including Delphi surveys. Clinical experts remain the most common stakeholder group involved. Overall, only 16% of the 259 COS studies published up to the end of 2016 have included participants from LMICs.

**Conclusion:**

This review highlights opportunities for greater public participation in COS development and the involvement of stakeholders from a wider range of geographical settings, in particular LMICs.

## Introduction

Measuring appropriate outcomes in clinical trials enables the benefits and harms of specific treatments to be compared between trials and allows decision makers, such as patients and clinicians, to be best informed in their choice. This is known as comparative effectiveness research (CER) [1]. Heterogeneity of outcome reporting in trials is, however, common, even in trials exploring the effects of the same intervention on a specific disease [2]. The lack of comparability that results from this causes waste in research [3,4]. This waste could be avoided with the development and systematic use of core outcome sets (COS) in clinical trials research, as demonstrated by the uptake in trials of a COS developed in rheumatoid arthritis [5]. A COS represents an agreed minimum set of outcomes that should be measured and reported in all trials for a specific health condition [2]. An element of the scope of the COS, to be defined, is whether the COS is to be applicable to all interventions or exclusively to specific intervention types. The use of COS would ensure all trials produce evidence that can be combined appropriately with the results of other trials, while not restricting the researchers’ ability to explore other, additional outcomes [2].

The purpose of the COMET (Core Outcome Measures in Effectiveness Trials) Initiative is to encourage the development, promotion and application of COS across all health areas. It does so by maintaining an up-to-date, online, publicly-accessible database of ongoing and published COS studies. Three systematic reviews have comprehensively searched for COS; the original review, conducted in 2013 [6], and two updates, one conducted in 2015 [7] and the second conducted in 2016 [8].

The 2016 update showed that COS had been developed for 13 of the world’s 25 most prevalent health conditions, leaving 12 of the 25 as being in need of COS development [8]. Eighty-two percent of the COS applicable to the world’s most prevalent health conditions involved participants from Europe and North America only. International participation in COS was highlighted as a key research gap. Representation of stakeholders from Africa and South America was particularly low in comparison to other continents, at only one and three studies (respectively). Further research is needed to establish how COS can be developed to have the greatest global relevance and applicability to the global burden of disease.

### Aims

The aims of the current study were to (i) update the systematic review [6–8] in order to identify any further studies where a COS has been developed; (ii) to describe the methodological approaches taken in these studies, (iii) to identify countries in which COS development participants are located and (iv) to highlight areas for future COS development and improvement.

## Methods

### Systematic review update

The methods used in this updated review followed the same approach used in the original review and in the previous two updates [6–8].

#### Study selection

**Inclusion and exclusion criteria:** As described in detail previously [6], studies were eligible for inclusion if they developed or applied methodology for determining which outcome domains or outcomes should be measured, or are important to measure, in clinical trials or other forms of health research. As noted in the 2016 update [8], by using the term `outcome' we are referring to something that occurs as a result of the specific health condition (e.g. diarrhoea) and by `outcome domain' we are referring to the grouping of individual outcomes (e.g. bowel function, which would include diarrhoea).

**Types of participants and interventions:** As described previously [6], studies were categorised as eligible if they reported the development of a COS, regardless of any restrictions by age, health condition or setting, which could be used to assess the effect of interventions for that condition.

#### Identification of relevant studies

In March 2017, we searched MEDLINE via Ovid and SCOPUS (including EMBASE) without language restrictions. The search identified studies that had been published or indexed between the previous systematic review update [8] in January 2016 and the end of December 2016. The multifaceted search strategy, developed for the original review using a combination of text words and index terms and combining three concepts of search terms that cover ‘randomised trial’, ‘systematic review’, ‘methodology’ and ‘outcomes’ [9] was used in the current update, with adaptations appropriate for each database (S1 Table). The search was deferred until March 2017 due to indexing issues that had been identified with the previous reviews [8], namely that studies published at the end of a particular year of interest had not been indexed in the database until the beginning of the following year and hence were not captured by the database search. We searched in March for this update to try to overcome this limitation. In addition to this database searching, we also completed hand searching activities as previously described [8]. We identified any studies that had been submitted directly to the COMET database. We also examined references cited in eligible studies and in ineligible studies that referred to or used a COS.

#### Selecting studies for inclusion in the review

Records from each database were combined and duplicates were removed. Titles and abstracts were read to assess eligibility of studies for inclusion in the review (stage 1). Full texts of potentially relevant articles were obtained to assess for inclusion (stage 2). Titles and abstracts were divided between reviewers (KD, NH, VS and PRW) who independently checked the title and abstract of each citation assigned to them and classified them as include, exclude or unsure. Where there was uncertainty in the classification of a record, a second reviewer was consulted. If agreement could not be achieved, the citation was referred to a fifth reviewer (SG). Full papers were divided between three reviewers (KD, NH and VS) for full-paper review. Reasons for exclusion at this stage were documented for articles judged to be ineligible.

#### Checking for agreement between the reviewers

During each stage of the review process, agreement between reviewers was assessed. Prior to independently assessing records, the four reviewers (KD, NH, VS and PRW) independently checked batches of abstracts and full papers to confirm consistency. This was repeated in batches of 10 until complete agreement was reached in three consecutive batches.

#### Checking for correct exclusion

Of the records that had been excluded on the basis of the title and abstract, full text papers were obtained for a 1% sample and a fifth reviewer (SG) assessed correct exclusion. If any studies were identified as being incorrectly excluded, further checking was performed within the other excluded records. Of the records that had been excluded after reading their full text papers, 5% were assessed for correct exclusion at that stage. In this review, no studies were excluded incorrectly.

#### Data extraction

As described in detail previously [6], data were extracted in relation to the study aim(s), health area, target population, interventions covered, methods of COS development and stakeholder groups involved. Data relating to the geographical locations of participants included in the development of COS were extracted for studies found in the current update, as was done in the previous reviews.

#### Data analysis and results

As previously [6], we used a narrative analysis and the results are presented descriptively. The results were analysed for the scope of their aims, intended use, population characteristics and intervention characteristics. The methods used, and stakeholders involved in selecting outcomes were also analysed. This update also included an analysis of the inclusion of participants from low and middle income countries (LMICs), as identified by the OECD, in these COS.

## Results

### Description of studies

**Fig 1:**
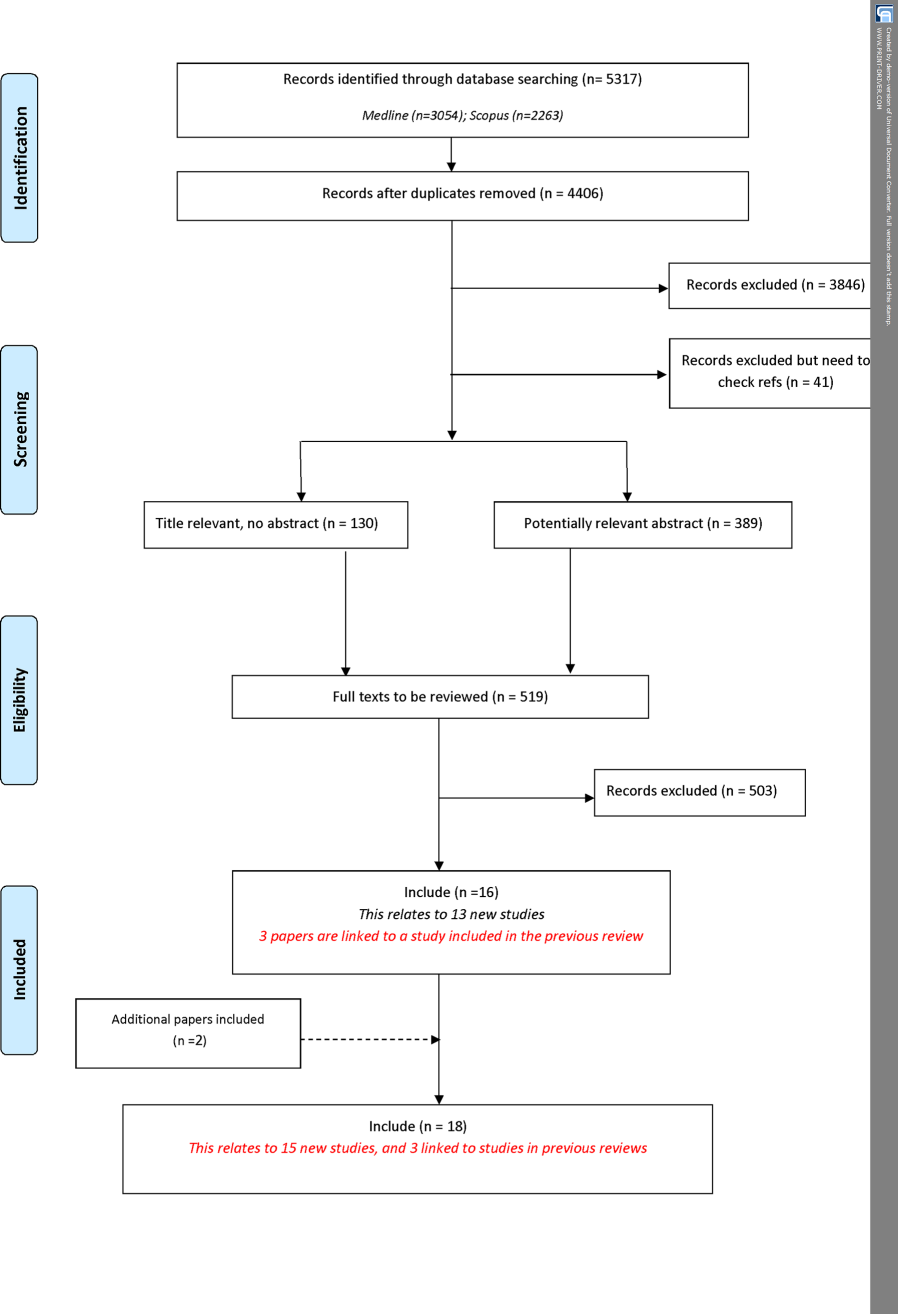
Identification of studies.

Following the removal of duplicates, 4406 citations were identified in the database search. A total of 3887 records were excluded during the title and abstract stage, and a further 503 were excluded following the assessment of full text papers (Fig 1). Table 1 provides a summary of the reasons for exclusion of papers at abstract and full text stage. Sixteen citations relating to 13 new studies met the inclusion criteria. In addition to the database search, two additional citations were identified as being eligible for inclusion in the review. In total, 18 reports relating to 15 new studies describing the development of 15 COS were added to the review during this update (S2 Table).

**Table 1:** Reasons for exclusion at full text stage.

Exclusion Categories of Full Text Stage	Number of Papers
Studies relating to how, rather than which, outcomes should be measured	21
Studies reporting the design/ rationale of single trial	1
Studies reporting the use of a COS	2
Systematic reviews of clinical trials	13
Systematic review of prognostic studies	2
Review/overview/discussion only, no outcome recommendations	37
Core outcomes/ outcome recommendations not made	63
Quality indicators–structure and/or process of care only	10
One outcome/ domain only	3
Instrument development	4
Recommendations by single author only	1
ICF Core set	0
Describes features of registry	9
Preclinical/ Early phase only (0, I, II)	5
Quantitative description	1
Value attributed to outcomes	0
Irrelevant	271
Assessed in previous review	0
Systematic review of outcomes used in studies	12
HRQL	3
Recommendations for clinical management in practice not research	31
Studies that elicit stakeholder group opinion regarding which outcome domains or outcomes are important	10
Ongoing studies	4

Three of the reports included in this updated are linked to COS studies that were reported in previous reviews [6,7]. One report is linked to a previously published preliminary COS, which has since been modified and endorsed [10]. Two further reports are linked to previously published COS, which have been revised to meet the current standards of outcome measure development [11,12]. All updates and revisions reported in the linked papers are reflected in the tables below.

### Included studies

#### Year of publication

**Fig 2:**
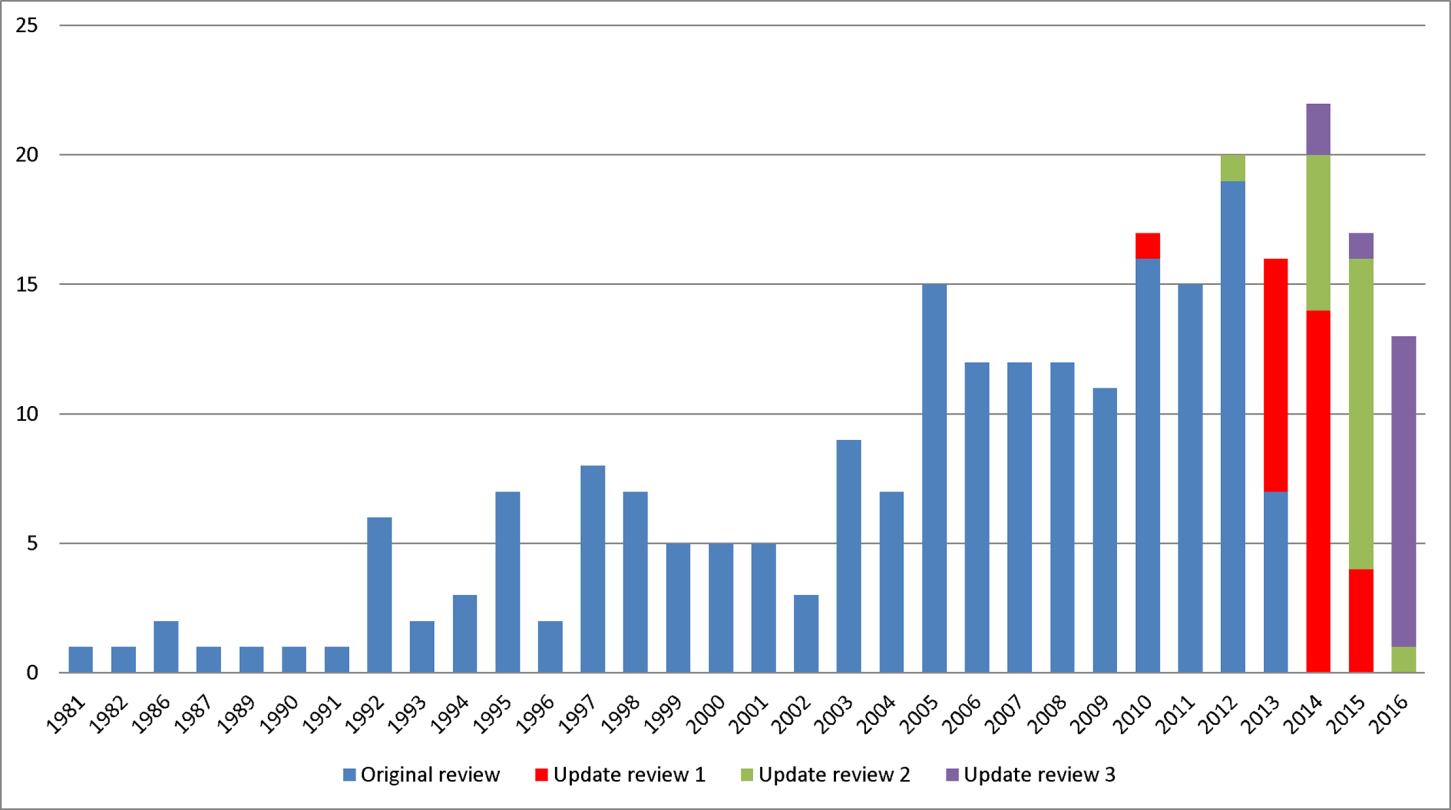
Year of first publication of each COS study (n = 259).

Our analysis of the year of first publication of each COS study included in the previous reviews has been updated to include the 15 new studies identified in this updated review (Fig 2). Of the 15 studies discussed here, 13 studies were published in 2015 or 2016, and two studies were published in 2014. Two studies were identified through reference checking of a COS study. One of these studies had been excluded from a previous review at the title and abstract screening stage because of the lack of COS information in the abstract [13]. The other study was identified through reference checking of related COS studies and had not come up in any of the previous review searches [14]. As referred to by the study authors, the scope of this COS, specifically the health condition, overlaps with earlier COS.

#### Scope of core outcome sets

The scope of published COS studies is summarised in Table 2 and includes 259 COS studies (describing the development of 309 COS) that were included in the three previous systematic reviews and the 15 new COS studies that have been added by this updated review. Please see S2 Table in supplementary material for the scope of the disease categories and names relevant to each of the 15 new COS. During this update, five studies that were judged to be COS in previous reviews were reviewed and excluded [15–19]. One was retrospectively deemed to refer to how to measure outcomes rather than what to measure [18] and four others were deemed to be composite end points rather than core outcome sets [15–17,19].

**Table 2:** The scope of included studies (n = 259).

	Update review 3 n (%)	Combined n (%)
**Study aims**		
Specifically considered outcome selection and measurement	10 (60)	142 (54)
Considered outcomes while addressing wider clinical trial design issues	5 (40)	117 (46)
**Intended use of recommendations**		
Clinical research	9 (60)	226 (87)
Clinical research and practice	6 (40)	33 (13)
**Population characteristics**		
Adults	6 (40)	36 (14)
Children	0 (0)	30 (12)
Adults and children	8 (53)	25 (10)
Older adults	0 (0)	3 (1)
Adolescents and young adults	1 (7)	1 (<1)
Not specified	0 (0)	164 (63)
**Intervention characteristics**		
All intervention types	8 (53)	39 (15)
Drug treatments	0 (0)	42 (16)
Surgery	4 (27)	25 (10)
Vaccine	0 (0)	2 (<1)
Rehabilitation	1 (7)	3 (1)
Exercise	0 (0)	3 (1)
Procedure	0 (0)	6 (2)
Device	1 (7)	4 (1)
Other	1 (7)	17 (7)
Not specified	0 (0)	118 (46)

#### Methods used to select outcomes

The methods used to develop the 15 new COS are presented in Table 3 alongside the methods used in the combined reviews [6–8]. The results show an increase in the use of mixed methods, including Delphi surveys.

**Table 3:** The methods used to develop COS (n = 259).

Main methods	Update review 3 n (%)	Combined n (%)
**Semi-structured group discussion only**		59 (23)
**Unstructured group discussion only**		18 (7)
**Consensus development conference only**		13 (5)
**Literature/systematic review only**	1 (7)	19 (7)
**Delphi only**		10 (4)
**Survey only**		3 (1)
**NGT only**		1 (<1)
**Mixed methods *(see descriptions below*)**^**1**^	12 (80)	116 (45)
*Delphi + another method(s)*	*9 (60)*	*48 (19)*
*Semi-structured group discussion + another method(s)*	*2 (13)*	*42 (16)*
*Consensus development conference + another method(s)*		*7 (3)*
*Literature/systematic review + another method(s)*	*1 (7)*	*14 (5)*
*NGT + another method(s)*		*4 (2)*
*Focus group + another method(s)*		*1 (<1)*
**No methods described**	2 (13)	20 (8)

^1^ Mixed methods studies are reported cumulatively. For example if they have been included in one subcategory they will be excluded from subsequent categories even if they apply.

#### Stakeholders involved in selecting outcomes

Table 4 lists the stakeholders that were involved in selecting outcomes for inclusion in the COS identified in this update and the combined reviews. Regarding the 259 published COS studies, 225 have provided details about the stakeholders who participated in the development process. Of these 225 studies, clinical experts have been involved in selecting outcomes for inclusion in 222 (99%) studies; this contrasts with public representatives, who have been included in only 62 (28%) studies.

**Table 4:** Participant groups involved in selecting outcomes for inclusion in COS (n = 225).

Participants category	Sub-category (not mutually exclusive)	Frequency of participants	
		Update review 3 n (%)^1^	Combined n (%)^2^
**Clinical experts**		**14 (93)**	**222 (99)**
	Clinical experts	14	131 (58)
	Clinical research expertise	2	87 (39)
	Clinical trialists/Members of a clinical trial network		11 (5)
	Others with assumptions		54 (24)
**Public representatives**		**8 (53)**	**62 (28)**
	Patients	8	45 (20)
	Carers	3	14 (6)
	Patient support group representatives		15 (7)
	Service users	1	3 (1)
**Non-clinical research experts**		**2 (13)**	**73 (32)**
	Researchers	2	37 (16)
	Statisticians		26 (12)
	Epidemiologists		14 (6)
	Academic research representatives		4 (2)
	Methodologists		11(5)
	Economists		4 (2)
**Authorities**		**0**	**47 (21)**
	Regulatory agency representatives		37 (16)
	Governmental agencies		13 (6)
	Policy makers		5 (2)
	Charities		1 (<1)
**Industry representatives**		**0**	**38 (17)**
	Pharmaceutical industry representatives		34 (15)
	Device manufacturers		3 (1)
	Biotechnology company representatives		1 (<1)
**Others**		**1 (7)**	**76 (34)**
	Ethicists		1 (<1)
	Journal editors		3 (1)
	Funding bodies		1 (<1)
	Yoga therapists/instructors		1 (<1)
	Members of health care transition research consortium	1	1 (<1)
	Others (besides known participants)		15 (7)
	Others with assumptions		54 (24)
**No details given**		**0 (0)**	**34 (15)**

^**1**^Studies providing details about participants groups involved in selecting outcomes (n) = 15

^**2**^Studies providing details about participants groups involved in selecting outcomes (n) = 225

Public representatives include patients, carers, health and social care service users and people from organisations who represent these groups. The degree of public participation within the development of the COS studies included in this updated review is described in Table 5. Seven of the eight studies that reported including public participants, provided details about their participation. Among studies that report public participation, levels of participation range from 15% [20] to 66% [21]. Where patients are involved, they tend to make up a greater percentage of the participants than in COS reported in previous reviews.

**Table 5:** Public participation detail where reported (n = 7).

	Methods used	Total number of participants	Number of public participants	% Public participants
1	Delphi (clinician only)	Round 1: 75	Round 1: 0	
		Round 2: 48	Round 2: 0	
	Delphi (mixed)^1^	Round 3: 61	Round 3: 24	39%
	Meeting (mixed)	16	2	15%
2	Delphi (mixed)	Round 1: 258	Round 1: 90	35%
		Round 2: 200	Round 2: 80	40%
		Round 3: 173	Round 3: 71	41%
	Meeting (clinician only)	33	0	
	Meeting (patient only)	9	9	
3	Meeting (clinician only)	16	0	
	Survey (clinician only)	16	0	
	Survey (patient only)	0	221	
4	Delphi (mixed)	Round 1: 228	Round 2: 150	66%
		Round 2: 208	Round 2: 135	65%
5	Delphi (mixed)	Round 1: 115	Round 1: 55	48%
		Round 2: 101	Round 2: 46	46%
		Round 3: 86	Round 3: 35	41%
6	Delphi (mixed)	Round 1: 195	Round 1: 97	50%
		Round 2: 165	Round 2: 87	53%
	Meeting (clinician only)^2^	61	0	
		35	0	
	Meeting (patient only)	14	14	
7*	Survey (patient only)	615	615	

1Patients brought in at round 3.

2 A second clinician meeting was held as consensus was not reached within the allotted time during the first clinician meeting

* Patient core set

### Countries involved in the development of COS

Table 6 lists the geographical locations, by continent, of the participants involved in developing the COS in this update and in the combined reviews, as reported by the included studies.

**Table 6:** Geographical locations of participants included in the development of each COS (n = 214).

Continents	Update review 3 n (%)	Combined n (%)
North America	6 (55)	167 (78)
Europe	10 (91)	167 (78)
Australasia	3 (27)	56 (26)
Asia	1 (9)	45 (21)
South America	1 (9)	24 (11)
Africa	1 (9)	15 (7)
No details provided	4 (27)	45 (17)

**Fig 3:**
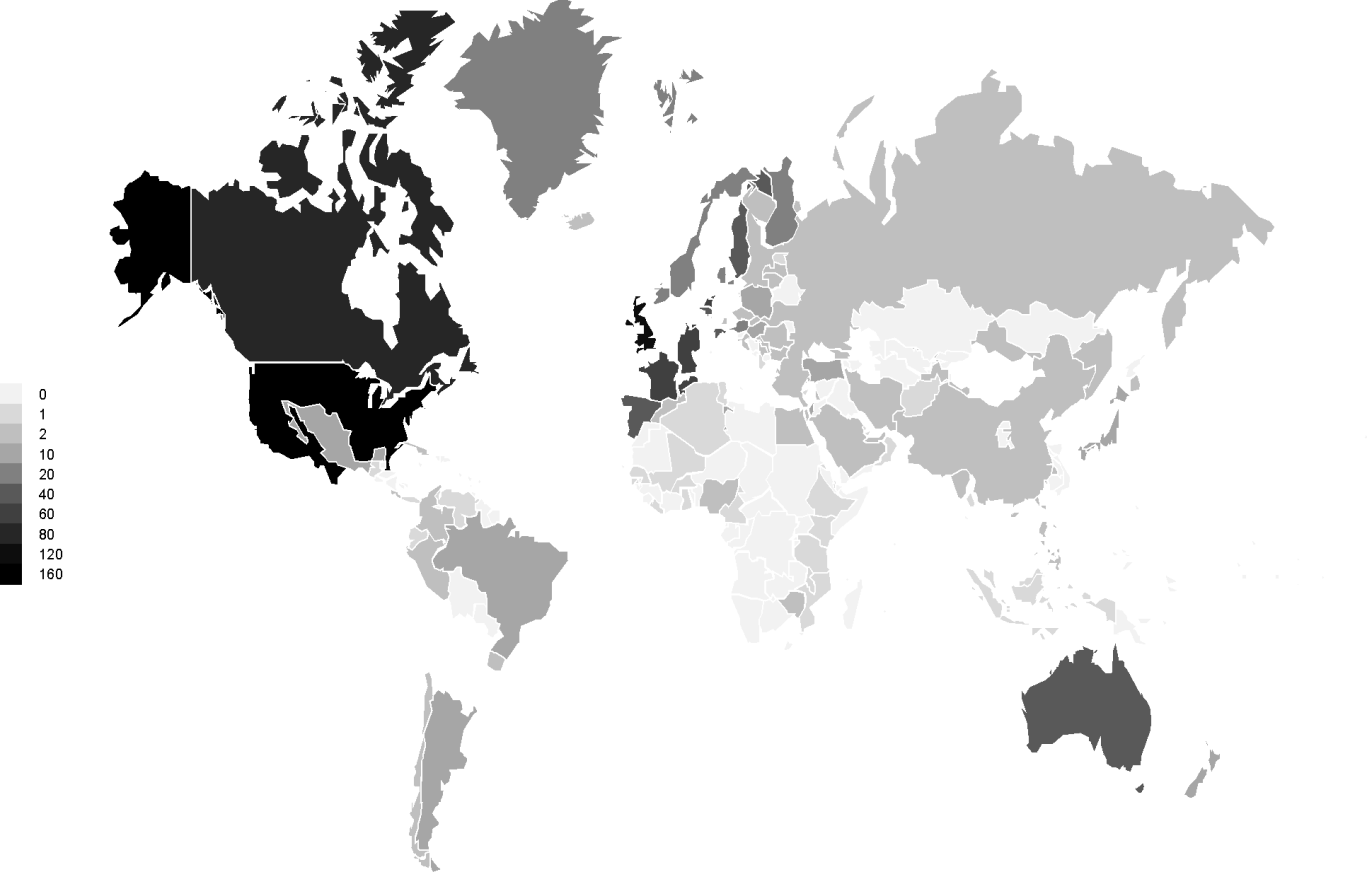
Number of COS studies that have included participants from each individual country throughout the world.

Fig 3 displays a choropleth map of the world, which uses differences in shading to indicate the number of COS studies that have been developed with the inclusion of participants from each country. Of the 259 COS studies published by the end of 2016, the countries which have been most involved in the COS development process are the United States of America (USA) (n = 161) and the United Kingdom (UK) (n = 124). There are 93 countries which are displayed on the map in the lightest shading; these represent the locations that have not been included in the development of any COS.

Of the 259 published COS studies, 41 (16%) have included participants from low and middle income countries (LMICs). These LMICs were identified according to the Organization for Economic Co-operation and Development (OECD) Development Assistance Committee (DAC) list of Official Development Assistance (ODA) recipients [22]. Table 7 presents a breakdown of the LMICs that have been involved in the development of COS.

**Table 7:** OECD DAC list of ODA recipient countries involved in the COS development process.

DAC^1^ list classifications	Number of COS involving participants	Countries
Least Developed Countries	4	Afghanistan, Burkina Faso, Ethiopia, Gambia, Liberia, Malawi, Mali, Mozambique, Nepal, Senegal, Tanzania
Other Low Income Countries	3	Kenya, Zimbabwe
Lower Middle Income Countries	17	Cameroon, Egypt, Georgia, Ghana, Guatemala, India, Indonesia, Morocco, Nigeria, Pakistan, Philippines, Sri Lanka, Ukraine
Upper Middle Income Countries	39	Albania, Algeria, Argentina, Brazil, Chile, China, Colombia, Costa Rica, Cuba, Ecuador, Iran, Lebanon, Malaysia, Mexico, Panama, Peru, Serbia, South Africa, Thailand, Tunisia, Turkey, Uruguay, Venezuela

1 Development Assistance Committee

## Discussion

This update to the previous three COMET Initiative systematic reviews has identified 18 publications relating to 15 new COS, suggesting that COS continue to be developed and published.

A range of resources are available on the COMET website to facilitate COS development. These include a core resource pack, plain language summaries and information about how to optimise the involvement of parents, young people and patient organisations in COS studies. This work has now been brought together in the COMET Handbook [23] to provide guidance on the development, application and implementation of COS.

Of the 259 COS studies published up to the end of 2016, the USA and the UK continue to be the most frequently represented countries by stakeholders involved. Use of the COMET website is continuing to increase, with the number of visitor locations reaching 175 countries in 2017 [24], however there are 96 countries that have not been included in the development of any COS. COS developed with globally rather than nationally located participants might improve the global applicability of clinical trials. To be comprehensive, a COS should be applicable in all relevant settings, including across different countries where appropriate. COS are more likely to be applicable within those countries that the participants in the development process are from. Therefore, if a COS is intended to have international applicability, this would have implications for how it is developed, who is involved, and the resources required for the development process. In particular, the developers should involve participants from countries where the prevalence or burden of the disease is high, but, with this in mind, it is worth noting that only 16% of COS developed to date have included participants from LMICs. Furthermore, COS developed with participants from multiple countries might improve the global applicability of the COS and, consequently, the global relevance and impact of the clinical trials that these are used in. A survey has recently been undertaken to identify the top priorities for trials methodological research in LMICs to inform further research and ultimately to improve clinical trials in these regions. The priority most commonly graded as critically important, amongst 400 participants from over 80 countries, was choosing appropriate outcomes to measure [25].

### Implications

The 15 new COS studies have been added to the COMET database, ensuring that it will remain up to date and continue to assist trialists, researchers, clinicians and others to design clinical trials, guidelines and systematic reviews. It will also remain a key source of information for those looking to develop a COS. Although reporting has improved, use of the COS-STAR guideline [26] will help further.

The updated review reported here highlights a continuing need to involve participants from a greater range of geographical locations in COS development, particularly from LMICs, in order to increase the global relevance of COS.

### Limitations

Gathering accurate data on the geographic location of participants is dependent on the reporting of this information, which was provided for over 70% of the COS studies in this review update. It is possible that there could be more COS involving participants from LMICs than reported here, although, there is no obvious reason why those reporting geographic information may differ with respect to LMIC involvement.

The abstracts reviewed in this study were reviewed by a single author only, in contrast to previous reviews where two reviewers reviewed each abstract. It is conceivable that some COS may have been missed at this stage of screening, however checks have been put in place to minimise incorrect exclusion, including batch checks and additional reviewers checking samples of excluded studies. The impact of this lack of identification on the usefulness of the COMET database is minimised, as the annual systematic review update is only one of a number of methods used to populate the COMET database, which also include database alerts and direct contact with COS developers throughout the year.

### Conclusions

In conclusion, this study has demonstrated the continuing development of new COS. The results show an increase in the use of mixed methodologies for COS development but suggest a need to push for greater public participation and the involvement of stakeholders from a broader range of countries, in particular LMICs. These efforts will increase the applicability of COS to global health and tackling the global burden of disease.

## Supporting information

S1 PRISMA Checklist PRISMA checklist for content of a systematic review. (DOC)

S1 Table Search strategy. (DOCX)

S2 Table Table of reports included in updated review (n = 18). (DOCX)
