# Supplementary material for: Choosing important health outcomes for comparative effectiveness research: An updated systematic review and involvement of low and middle income countries
Source: PLoS One. 2018 Feb 13;13(2):e0190695. doi: 10.1371/journal.pone.0190695 (PMC5810981; doi:10.1371/journal.pone.0190695)
Supplement: S2 Table — (DOCX) [file pone.0190695.s003.docx]

**S2 Table.** Table of reports included in updated review (n=18)

| **Study** | **Disease category** | **Disease name** |
| --- | --- | --- |
| Al Wattar 2017** [[1](#_ENREF_1)] | Pregnancy and Childbirth | Epilepsy in Pregnancy |
| Audige 2016 [[2](#_ENREF_2)]^1^** | Orthopaedics & trauma | Arthroscopic Rotator Cuff Repair |
| Coulman 2016 [[3](#_ENREF_3)]** | Endocrine & metabolic | Obesity |
| Crabb 2016 [[4](#_ENREF_4)]* | Endocrine & metabolic | Alcoholic Hepatitis |
| Deyo 2015 [[5](#_ENREF_5)]* | Orthopaedics & trauma | Chronic Low Back Pain |
| Fair 2016 [[6](#_ENREF_6)]** | Other | Adolescents and young adults with chronic physical and medical conditions |
| Gerritsen 2016 [[7](#_ENREF_7)]** | Cancer | Pancreatic cancer |
| Helliwell 2016 [[8](#_ENREF_8)]** | Rheumatology | Polymyalgia rheumatica (PMR) |
| Ismail 2016 [[9](#_ENREF_9)]** | Eyes & vision | Glaucoma |
| Khanna 2015 [[10](#_ENREF_10)]** | Lungs & airways | Interstitial lung disease |
| Maahs 2016 [[11](#_ENREF_11)]** | Endocrine & metabolic | Type I Diabetes |
| Major 2016 [[12](#_ENREF_12)]^2^* | Rehabilitation | Critical Illness |
| McNair 2016 [[13](#_ENREF_13)]** | Cancer | Colorectal Cancer |
| Milman 2017 [[14](#_ENREF_14)]** | Heart & circulation | ANCA associated vasculitis |
| Myatt 2014 [[15](#_ENREF_15)]* | Pregnancy and Childbirth | Pre-eclampsia |
| Noble 2016 [[16](#_ENREF_16)]** | Neurology | Epilepsy |
| Orbai 2016 [[17](#_ENREF_17)]** | Rheumatology | Psoriatic arthritis |
| Pinder 2015 [[18](#_ENREF_18)]* | Orthopaedics & trauma | Scaphoid nonunion |

*^1^ COS for adverse events*

*^2^ COS developed for clinical practice, however author confirmed that it is applicable and usable for research*

** Considered outcomes while addressing wider clinical trial design issues*

*** Specifically considered outcome selection and measurement*

**References**

1. Al Wattar BH, Tamilselvan K, Khan R, Kelso A, Sinha A, Pirie AM, et al. Development of a core outcome set for epilepsy in pregnancy (E-CORE): a national multi-stakeholder modified Delphi consensus study. [BJOG: An International Journal of Obstetrics and Gynaecology.](https://www.ncbi.nlm.nih.gov/pubmed/27860117) 2017;124(4):661-667.
2. Audigé L, Flury M, Müller AM; ARCR CES Consensus Panel, Durchholz H. Complications associated with arthroscopic rotator cuff tear repair: definition of a core event set by Delphi consensus process. Journal of Shoulder and Elbow Surgery. 2016;25(12):1907-17.
3. Coulman KD, Hopkins J, Brookes ST, Chalmers K, Main B, Owen-Smith A, et al. A Core Outcome Set for the Benefits and Adverse Events of Bariatric and Metabolic Surgery: The BARIACT Project. PLoS Medicine. 2016;13(11):e1002187.
4. Crabb DW, Bataller R, Chalasani NP, Kamath PS, Lucey M, Mathurin P, et al. Standard Definitions and Common Data Elements for Clinical Trials in Patients With Alcoholic Hepatitis: Recommendation From the NIAAA Alcoholic Hepatitis Consortia. Gastroenterology. 2016;150(4):785-90.
5. Deyo, R.A., et al., Report of the NIH Task Force on Research Standards for Chronic Low Back Pain. Journal of Pain. 2014;15(6):569-85.
6. Fair C, Cuttance J, Sharma N, Maslow G, Wiener L, Betz C, et al. International and interdisciplinary identification of health care transition outcomes. JAMA Pediatrics. 2016;170(3):205-11.
7. Gerritsen A, Jacobs M, Henselmans I, van Hattum J, Efficace F, Creemers GJ, et al. Developing a core set of patient-reported outcomes in pancreatic cancer: A Delphi survey. European journal of cancer. 2016;57:68-77.
8. Helliwell T, Brouwer E, Pease CT, Hughes R, Hill CL, Neill LM, et al. Development of a Provisional Core Domain Set for Polymyalgia Rheumatica: Report from the OMERACT 12 Polymyalgia Rheumatica Working Group. The Journal of rheumatology. 2016;43(1):182-6.
9. Ismail R, Azuara-Blanco A, Ramsay CR. Consensus on outcome measures for glaucoma effectiveness trials: Results from a delphi and nominal group technique approaches. Journal of Glaucoma. 2016;25(6):539-546.
10. Khanna D, Mittoo S, Aggarwal R, Proudman SM, Dalbeth N, Matteson EL, et al. Connective Tissue Disease-associated Interstitial Lung Diseases (CTD-ILD) - Report from OMERACT CTD-ILD Working Group. The Journal of rheumatology. 2015; 42(11):2168-71.
11. Maahs DM, Buckingham BA, Castle JR, Cinar A, Damiano ER, Dassau E, et al. Outcome measures for artificial pancreas clinical trials: A consensus report. Diabetes Care. 2016;39(7):1175-9.
12. Major ME, Kwakman R, Kho ME, Connolly B, McWilliams D, Denehy L, et al. Surviving critical illness: What is next? An expert consensus statement on physical rehabilitation after hospital discharge. Critical Care. 2016;20:354.
13. McNair AG, Whistance RN, Forsythe RO, Macefield R, Rees J, Pullyblank AM, et al. Core Outcomes for Colorectal Cancer Surgery: A Consensus Study. PLoS Medicine. 2016;13(8):e1002071.
14. Milman N, Boonen A, Tugwell P, Merkel PA; OMERACT Vasculitis Working Group. Clinicians’ perspective on key domains in ANCA-associated vasculitis: a Delphi exercise. Scandinavian Journal of Rheumatology. 2017;46(2):112-7.
15. Myatt L, Redman CW, Staff AC, Hansson S, Wilson ML, Laivuori H, et al. Strategy for Standardization of Preeclampsia Research. Hypertension. 2014;63(6):1293-301.
16. Noble AJ, Marson, AG. Which outcomes should we measure in adult epilepsy trials? The views of people with epilepsy and informal carers. Epilepsy & Behavior. 2016;59:105-110.
17. Orbai AM, de Wit M, Mease P, Shea JA, Gossec L, Leung YY, et al. International patient and physician consensus on a psoriatic arthritis core outcome set for clinical trials. Annals of the Rheumatic Diseases. 2017;76(4):673-80.
18. Pinder RM, Brkljac M, Rix L, Muir L, Brewster M. Treatment of Scaphoid Nonunion: A Systematic Review of the Existing Evidence. The Journal of Hand Surgery. 2015;40(9):1797-1805.
